# Magnolol exerts anti-inflammatory effect in the animal model of rheumatoid arthritis

**DOI:** 10.1515/biol-2025-1352

**Published:** 2026-09-03

**Authors:** Guifu Qin, Siqi Li, Chong Chen, Yalong Qin, Zhiqin Ye, Tingting Pen, Qin Zhuo, Yuan Liang

**Affiliations:** Department of Rheumatology, Hubei Provincial Hospital of Traditional Chinese Medicine, Wuhan, 430061, Hubei, China; Hubei Key Laboratory of Theory and Application Research of Liver and Kidney in Traditional Chinese Medicine, Wuhan, Hubei, China; Department of Hematopathology, Hubei Provincial Hospital of Traditional Chinese Medicine, Wuhan, 430000, Hubei, China; Department of Acupuncture and Moxibustion, Department of Rehabilitation, Yidu Hospital of Traditional Chinese Medicine, Yichang, 443300,Hubei, China; Department of Geriatrics, Wuhan Hospital of Traditional Chinese Medicine, Wuhan, 430000, China

**Keywords:** magnolol, inflammation, MH7A cells, CIA rats, RA

## Abstract

As a typical inflammatory arthritis, rheumatoid arthritis (RA) reduces the life quality of patients from all over the world. Magnolol has been reported to exert the anti-inflammation role in several diseases. Here, we aim to investigate the effect of magnolol on the inflammatory response *in vivo* and *in vitro*. Indicators like arthritic index and paw weight were measured after collagen-induced arthritis (CIA) induction and magnolol treatment. The tissues of the rats’ left hind paws were subjected to hematoxylin & eosin staining to observe the morphological changes. The serum inflammatory cytokines and MH7A cells were evaluated by enzyme‐linked immunosorbent and real time quantitative polymerase chain reaction. Cell viability assay and flow cytometry were prepared for evaluating the viability and apoptosis of MH7A cells, respectively. Magnolol alleviated the paw inflammation and joint swelling in CIA rat model. CIA-induced upregulation of the serum inflammatory markers was also significantly reduced by magnolol in polyarthritis rat model. For *in vitro* experiments, magnolol treatment enhanced the viability and decreased apoptosis of tumor necrosis factor-alpha (TNF-α)-stimulated MH7A cells. Inflammatory cytokines were also downregulated by magnolol in TNF-α-stimulated MH7A cells. Magnolol alleviated RA development by inhibiting inflammatory response in CIA-induced rat model or TNF-α-treated MH7A cells.

## 1 Introduction

Rheumatoid arthritis (RA), a systemic and autoimmune disease, affects approximately 1 % of the global population, [1], [2], [3]. Several studies suggested that RA is closely associated with the risk factors for other comorbidities, including hypothyroidism and atherosclerosis [4], [5], [6]. RA patients often suffer from joint stiffness and swelling [7], which is caused by cartilage degradation and synovial inflammation [8], 9]. Currently, drug therapy has become a main treatment for RA, Non-steroidal anti-inflammatory drugs and disease-modifying anti-rheumatic drugs are widely utilized therapeutic agents in the clinical management of RA [10], 11]. However, their clinical applications are still limited due to the accompanying side effects like gastrointestinal disorders, humoral disorders, and immune deficiency [12]. Therefore, natural medicine with less side effects and more effective functions for RA treatment is in urgent demand.

Magnolol, extracted from Chinese medicinal herb *Magnolia officinalis,* has long been used for the treatment of various diseases including fever, headache, asthma, anxiety, and diarrhea [13], [14], [15]. Moreover, magnolol was reported to possess anti-inflammatory [16], [17], [18], anti-oxidative [19], 20], antimicrobial [21], and neuroprotective [22] properties, and its anti-inflammatory effect has been revealed in several diseases, including steatosis [23], diabetic periodontitis [24], osteoarthritis [25], endometritis [26], and intracerebral haemorrhage [27]. Notably, magnolol participated in the regulation of inflammatory arthritis and sereved as a potential anti-arthritic agent, which inhibits the expression of inflammation associated genes via nuclear factor-kappa B (NF-κB) and mitogen-activated protein kinases (MAPKs) pathways [28]. However, its anti-inflammatory role and mechanism in TNF-α-stimulated human RA synoviocyte MH7A cell lines and collagen-induced arthritis (CIA) rat model have not been illustrated yet.

Here, we investigated the effect of magnolol on the inflammatory response in TNF-α-stimulated human RA synoviocyte MH7A cell lines and CIA rat model, which further confirmed the role of magnolol in RA development and provided a novel research strategy for RA treatment.

## 2 Materials and methods

### 2.1 Animal model

Twenty-four female Lewis rats (five-week-old weight 180–220 g) were obtained from Hubei Bainte Biotechnology Co., Ltd. maintained in their cages under standard laboratory conditions (a constant temperature of 22–24 °C, 12 h light/dark cycle, and humidity of 40–70 %) and given free access to food and water. Rats were then randomly divided into four groups (*n* = 6): Sham + dimethyl sulfoxide (DMSO), Sham + magnolol, CIA + DMSO, CIA + magnolol. Collagen-induced arthritis (CIA) was caused in CIA + DMSO and CIA + magnolol groups by administering collagen type II as previously described [29]. Specifically, type II collagen solution (2 g/ml) was emulsified in the same volume of complete Freund’s adjuvant. Each rat was injected subcutaneously with 200 μg of the emulsion at the bottom of the tail. On day 21, those rats received second injection to establish CIA model. Rats in the sham groups (Sham + DMSO and Sham + magnolol) were injected with equal volume saline. Rats in magnolol groups (Sham + magnolol and CIA + magnolol) were randomly administered intraperitoneally with magnolol (10 mg/kg/day) for four weeks. On day 35, serum samples were collected, and all rats were euthanized via an intraperitoneal injection of pentobarbital sodium (150 mg/kg), and the left hind paws were prepared for the following experiments. Sample size was justified *a priori* through power analysis, confirming adequate statistical power to detect predefined meaningful effect sizes as described previously [30], 31]. The animal experiments were conducted by researchers blinded to the animal group allocation.

**Ethical approval:** The research related to animal use has been complied with all the relevant national regulations and institutional policies for the care and use of animals, and has been approved by the Animal Care and Use Committee of Hubei Bainte Biotechnology Co., Ltd. (BNT-2024-169; August 3, 2024).

### 2.2 Cell culture

Human RA synoviocyte MH7A cell line was purchased from American Type Culture Collection (ATCC, Maryland, USA) and cultured in DMEM containing 10 % FBS, penicillin (100 U/mL) and streptomycin (100 μg/mL) and maintained overnight at 37 °C with 5 % CO_2_ until confluency.

### 2.3 Cell treatment

To mimic pathological situation *in vitro*, cells were stimulated with 5, 10, 15 or 20 ng/mL of TNF‐α (MedChemExpress, Shanghai, China) for 6 h. Magnolol was also obtained from MedChemExpress and dissolved in DMSO (MedChemExpress). MH7A cells were then treated with different doses of magnolol (0.25, 0.75, 7.5, and 25 µg/mL) for 12 h. Subsequently, 10 ng/mL of TNF‐α and 7.5 µg/mL of magnolol were selected for following study.

### 2.4 Hematoxylin & eosin (H &E) staining

After the euthanasia, the left hind paws of the rats were immediately fixed in 4 % paraformaldehyde overnight, embedded with paraffin, and sliced into 5 μm thick tissue sections. Subsequently, the pathological improvement was assessed through staining the tissue sections with hematoxylin and eosin. Finally, photos were captured using an OLYMPUS microscope (Shanghai Optical Instrument Factory, Shanghai, China).

### 2.5 Enzyme‐linked immunosorbent (ELISA)

Before the euthanasia, serum samples of rats were collected to assess the levels of inflammatory cytokines. Rat IL‐6, IL-1β, MMP-1, MMP-3 ELISA kits (Shanghai Genetimes technology Inc., Shanghai, China) were prepared for detecting the levels of IL‐6, IL-1β, MMP‐1, and MMP‐3 in the rat serum Table 1.

**Table 1:** Experimental design 1.

Group	Treatment	Experiments
Sham + DMSO	Injection of saline and DMSO	Photographing, HE staining, qPCR and ELISA analyses
Sham + magnolol	Injection of saline and magnolol	Photographing, HE staining, qPCR and ELISA analyses
RA + DMSO	Injection of complete Freund’s adjuvant containing type II collagen solution and DMSO	Photographing, HE staining, qPCR and ELISA analyses
RA + magnolol	Injection of complete Freund’s adjuvant containing type II collagen solution and magnolol	Photographing, HE staining, qPCR and ELISA analyses

### 2.6 Real time quantitative polymerase chain reaction (qPCR)

After the indicated treatment, MH7A cells were used to isolate the total RNA and then reversely transcribed into cDNA using a PrimeScript™ RT Reagent Kit (Takara, Dalian, China). RT‐qPCR of inflammatory markers was then performed with a SYBR Premix Ex Taq II kit (Takara) on the ABI7500 quantitative PCR instrument (ABI Company, Oyster Bay, NY). The PCR program included an activation step at 95 °C for 30 s followed by 40 cycles of 5 s at 95 °C and 34 s at 60 °C. GAPDH was regarded as the internal reference and the relative mRNA expression was calculated using the 2^−ΔΔCt^ method. The experiment was repeated three times to obtain the mean value Table 2.

**Table 2:** Experimental design 2.

Group	Treatment	Experiments
Con + DMSO	Treated with saline and DMSO	CCK-8, flow cytometry, qPCR and ELISA analyses
Con + magnolol	Treated with saline and magnolol	CCK-8, flow cytometry, qPCR and ELISA analyses
TNF-α+DMSO	Treated with TNF-α and DMSO	CCK-8, flow cytometry, qPCR and ELISA analyses
TNF-α+magnolol	Treated with TNF-α and magnolol	CCK-8, flow cytometry, qPCR and ELISA analyses

### 2.7 Cell viability assays

MH7A cells were seeded in 96-well plate at 6 × 10^3^ cells/well, then 0.5 mg/mL of 3-(4,5-dimethylthiazol-2-yl)-2,5-diphenyl tetrazolium bromide (MTT) solution (Sigma-Aldrich, MO) was added. After the incubation at for 2 h at 37 °C, cells were then pelleted and lysed in DMSO (100 mL) and the absorbance at 550 nm measured on a microplate reader Table 3.

**Table 3:** ELISA kits information.

Proteins	Samples	Number	Source
IL-6	MH7A	ab178013	Abcam
IL-6	Rat’s serum	ab234570	Abcam
IL-1β	MH7A	ab214025	Abcam
IL-1β	Rat’s serum	ab100768	Abcam
MMP-1	MH7A	ab215083	Abcam
MMP-1	Rat’s serum	CB10464-Ra	COIBO BIO
MMP-3	MH7A	ab269371	Abcam
MMP-3	Rat’s serum	ab270216	Abcam

### 2.8 Flow cytometry

Treated MH7A cells were harvested and washed with PBS. After cells were resuspended, they were added with 5 μl of FITC Annexin V and 5 μl of PI and incubated for nearly 15 min at room temperature in the dark to analyze the cell apoptosis using flow cytometry with an Annexin V/PI apoptotic detection kit (Beyotime Institute of Biotechnology).

### 2.9 Statistical analysis

Prior to formal statistical analyses, normality testing of continuous variables was performed using the Shapiro-Wilk test. Homogeneity of variance across experimental groups was assessed via Levene’s test to ensure the robustness of between-group comparisons. The data were processed using SPSS 21.0 statistical software (IBM, Corporation, Armonk, NY, USA) and shown as mean ± SD Data within multiple groups is analyzed using one-way analysis of variance (ANOVA) and Tukey’s s *post hoc* test. *p* < 0.05 indicated that the difference is statistically significant, and the exact p-value was shown in results.

## 3 Results

### 3.1 Magnolol inhibits the CIA-induced polyarthritis development in the rats

**Figure 1:**
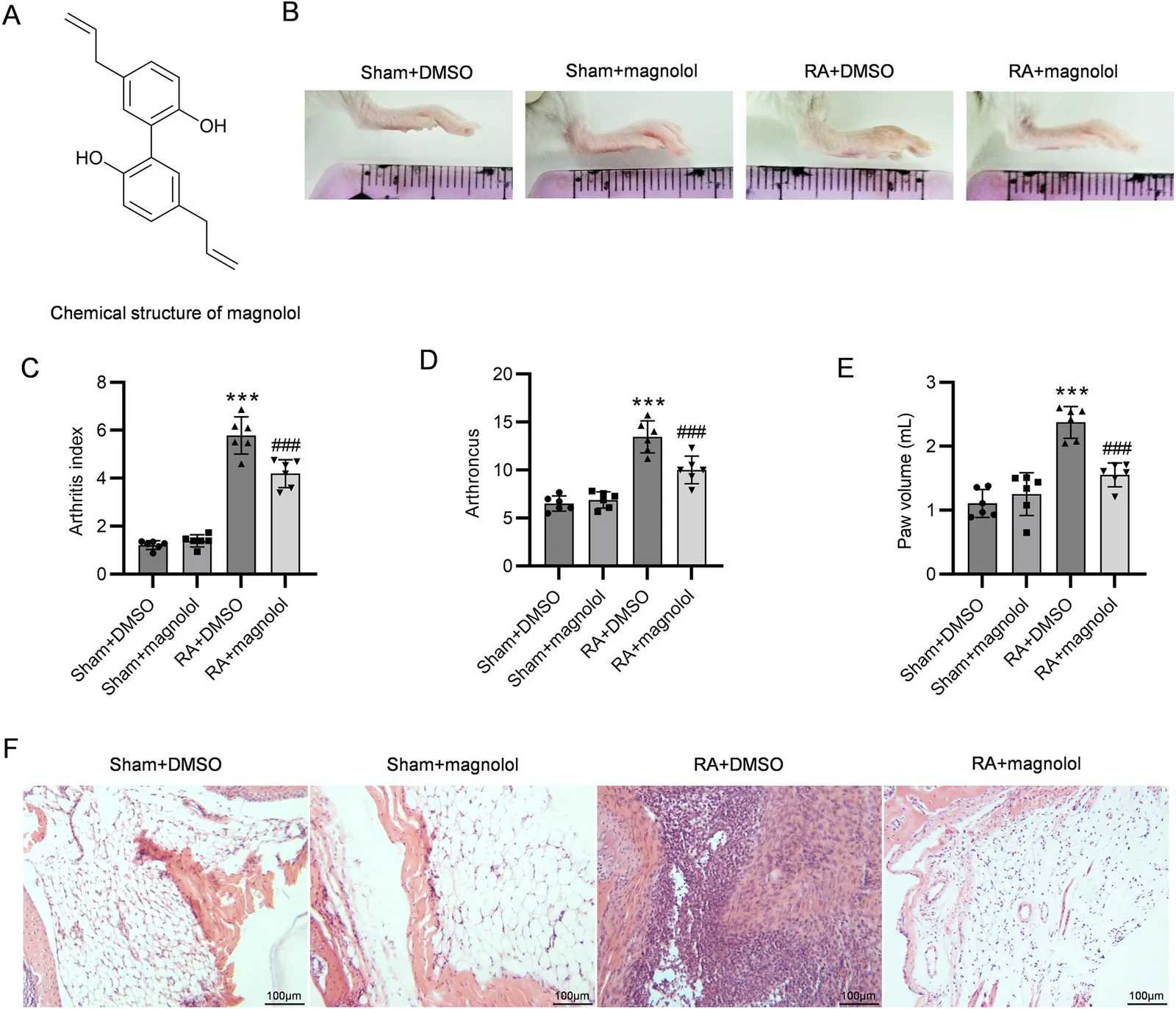
Magnolol inhibits the development and progression of CIA rats. (A) The structure of magnolol was shown. **(B)** CIA-induced paw inflammation and joint swelling was observed in Sham + DMSO, Sham + magnolol, RA + DMSO, RA + magnolol groups. **(C–E)** The arthritis index, arthroncus, and paw volume were examined in Sham + DMSO, Sham + magnolol, RA + DMSO, RA + magnolol groups. **(F)** HE staining was used to examine the pathological condition in Sham + DMSO, Sham + magnolol, RA + DMSO, RA + magnolol groups. *p < 0.05, **p < 0.01, ***p < 0.001.

The structure of magnolol was shown in Figure 1A. After the CIA induction, rats in the CIA groups presented inflamed paws, and swollen small joints, compared with those in sham-operated groups. Magnolol treatment significantly alleviated those phenomena (Figure 1B). Consistently, the arthritis index, arthroncus, and paw volume were all significantly decreased in the CIA rats treated with magnolol, compared with that in CIA + DMSO (Figure 1C–E). According to the results of the HE staining, CIA rats were infiltrated with inflammatory cells and showed increased synovial hyperplasia and bone destruction. While magnolol treatment significantly inhibited inflammatory cell infiltration, synovial hyperplasia, and cartilage damage in CIA rats, indicating that magnolol can improve the pathological condition of CIA rats (Figure 1F).

### 3.2 Magnolol alleviates the inflammatory response in CIA rats

**Figure 2:**
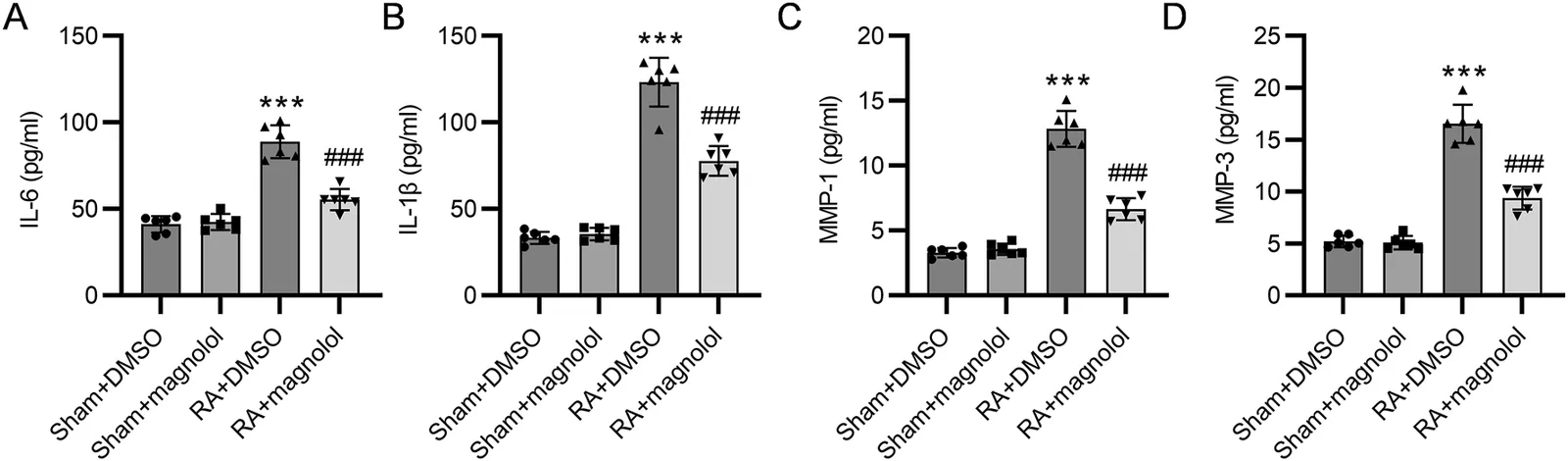
Magnolol alleviates the inflammatory response in CIA rats. **(A–D)** The serum levels of inflammatory markers were evaluated by ELISA in Sham + DMSO, Sham + magnolol, RA + DMSO, RA + magnolol groups. **p < 0.01, ***p < 0.001.

As shown in Figure 2, the CIA induction obviously increased serum levels of inflammatory cytokines (IL-6, IL-1β, MMP-1 and MMP-3) compared to the sham operated group. However, the levels of inflammatory cytokines were lowered in CIA + magnolol group relative to those in CIA + DMSO group, suggesting an inhibitory effect of magnolol on inflammatory response in CIA-induced rats (Figure 2A–D).

### 3.3 Magnolol relieves the dysfunction induced by TNF-α in MH7A cell lines

**Figure 3:**
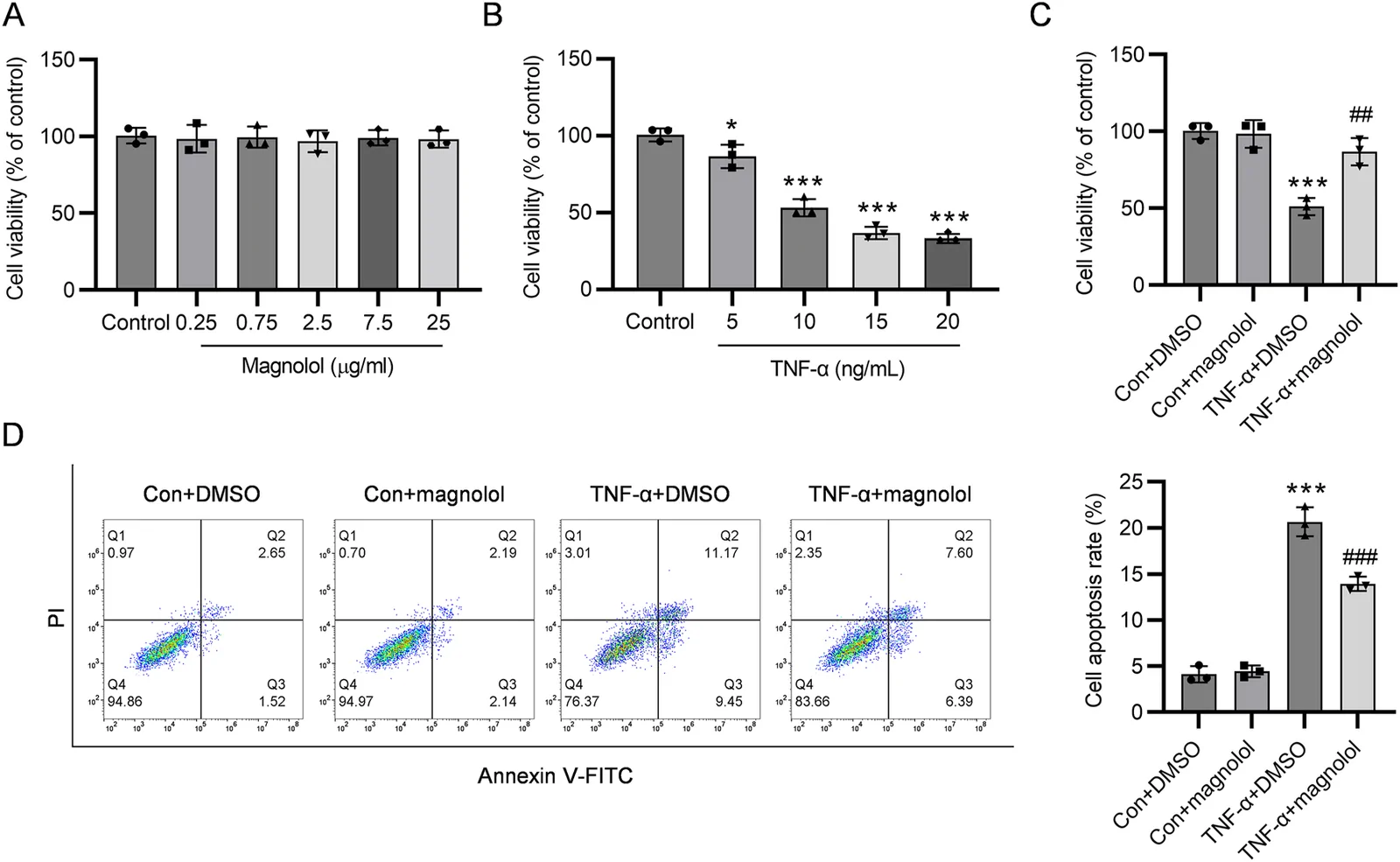
Magnolol relieves the dysfunction induced by TNF-α in MH7A cell lines. (A) The cell viability of human RA synoviocyte MH7A cell lines was evaluated by MTT assay after the treatment of magnolol at different concentrations (0.25, 0.75, 7.5, and 25 µg/mL). **(B)** The cell viability of human RA synoviocyte MH7A cell lines was evaluated by MTT assay after the treatment of TNF-α at different concentrations (5, 10, 15, and 20 ng/mL). **(C)** The viability of human RA synoviocyte MH7A cell lines was evaluated by MTT assay in con + DMSO, con + magnolol, TNF-α + DMSO, and TNF-α + magnolol groups. **(D)** Flow cytometry revealed that the cell apoptosis rate in con + DMSO, con + magnolol, TNF-α + DMSO, and TNF-α + magnolol groups. *p < 0.05, **p < 0.01, ***p < 0.001.

After confirming the function of magnolol *in vivo*, we further detected the effect of magnolol *in vitro*. The viability of TNF-α-stimulated MH7A cell lines was then evaluated by MTT assay, and we found that the cell viability had no significant changes after the treatment of magnolol at different concentrations (0.25, 0.75, 7.5, and 25 µg/mL) (Figure 3A). Inversely, the viability of TNF-α-stimulated MH7A cell lines gradually decreased with the accumulation of TNF-α concentration, indicating its ability to induce cellular damage (Figure 3B). MTT assay further revealed that the viability of TNF-α-stimulated human RA synoviocyte MH7A cell lines was significantly increased by magnolol, compared with that in TNF-α + DMSO group (Figure 3C). Flow cytometry revealed that the cell apoptosis rate had no significant change in the groups without TNF-α treatment. While TNF-α increased the apoptosis, and magnolol further reversed the changes and decreased the apoptosis of TNF-α-stimulated MH7A cell lines (Figure 3D and E).

### 3.4 Magnolol alleviates the inflammatory response in TNF-α-treated MH7A cell lines

**Figure 4:**
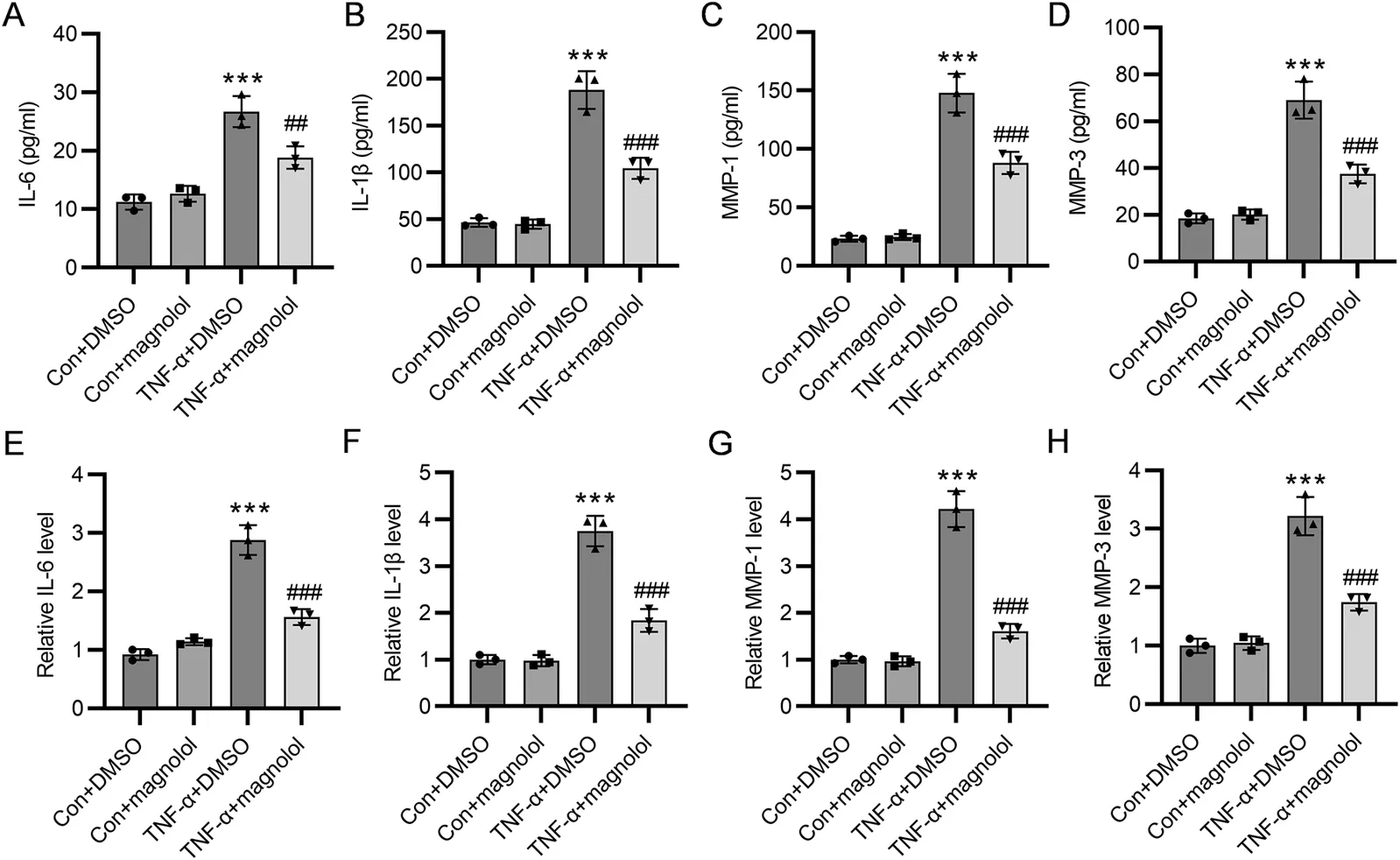
Magnolol alleviates the inflammatory response in TNF-α-treated MH7A cell lines. **(A–D)** ELISA was conducted to examine the levels of inflammatory markers in con + DMSO, con + magnolol, TNF-α + DMSO, and TNF-α + magnolol groups. **(E–H)** IL-6, IL-1β, MMP-1 and MMP-3 expression levels were examined by RT-qPCR in con + DMSO, con + magnolol, TNF-α + DMSO, and TNF-α + magnolol groups. *p < 0.05, **p < 0.01, ***p < 0.001.

ELISA was conducted to examine the levels of inflammatory cytokines in con + DMSO, con + magnolol, TNF-α+DMSO, and TNF-α+magnolol groups. As shown in Figure 4A–D, the concentration of inflammatory cytokines was upregulated by TNF-α treatment. However, magnolol partially reversed the changes. Consistently, the mRNA levels of those inflammatory markers were higher in TNF-α+DMSO group than those in con + DMSO group. Particularly, magnolol downregulated expression levels of the inflammatory markers in TNF-α-treated MH7A cell lines (Figure 4E–H).

## 4 Discussion

Severe joint pain and dysfunction in RA patients were commonly caused by the destruction of cartilage and bone, which is influenced by chronic arthritic inflammation and synovial tissue hyperplasia [32]. Pathologically, persistent inflammation and excessive oxidative stress injury act as critical pathological drivers [33], [34], [35], synergistically contributing to progressive cartilage degradation, bone erosion, and irreversible joint destruction in patients with RA. As the main effector cells of synovitis, synoviocytes play a crucial role in RA development [36]. Increasing studies showed that synoviocytes can be stimulated by TNF‐α [37], a master cytokine that essentially triggers inflammation and joint destruction in synoviocytes [38], and TNF‐α-treated synoviocytes are regarded as perfect *in vitro* model to mimic the characteristics of RA [39], 40]. Moreover, several studies suggested that TNF‐α treatment significantly induced the apoptosis of MH7A cells [41], 42]. CIA rat is always helpful to study possible therapeutic strategies for polyarthritis *in vivo* [43], 44]. Thus, in this study, the effect of magnolol on RA synoviocyte and polyarthritis rats was studied by stimulating MH7A cell lines with TNF-α and inducing rats with CIA, respectively.

Traditional Chinese Medicine formula-based therapies for RA have gained growing global acceptance in recent years, owing to their favorable safety profile and proven clinical efficacy. For example, the anti-rheumatoid arthritis effect of flavonoids of *Polygonum orientale* [45], the adlay seed extract [46], and paeoniflorin [47] etc. all has been widely reported. In addition, xuetongsu was reported to alleviate synovial inflammation by affecting macrophages and osteoclasts in rheumatoid arthritis [7], 48]. Notably, magnolol has been reported to have many properties including anti-inflammatory, and its anti-arthritic effect also has been shown in IL-1β-stimulated fibroblast‐like synoviocytes by previous study [28]. In detail, magnolol inhibited the release of cytokines in IL-1β-stimulated cells by inactivating NF-κB signaling and MAPK signaling. Additionally, administration of magnolol at a dose of 100 mg/kg for 16 consecutive days resulted in significant reductions in limb swelling and paw volume, as well as marked attenuation of leukocyte infiltration and synovitis. Unlike this, we here investigate its role in TNF-α-stimulated human RA synoviocyte MH7A cell lines and collagen-induced RA rats. Particularly, we found that magnolol alleviated CIA-induced paw inflammation and joint swelling and decreased the levels of inflammatory cytokines, suggesting its inhibitory role in the inflammatory response of collagen-induced RA rats. Consistently, magnolol reversed the effect of TNF-α treatment and promoted the cell viability and inhibited cell apoptosis of TNF-α-stimulated MH7A cell lines. The levels of inflammatory markers were upregulated by TNF-α treatment in MH7A cell lines. However, magnolol partially reversed the changes. Future study could focus on the possible molecular pathways that influence the effect of magnolol in TNF-α-stimulated human RA synoviocyte MH7A cell lines and collagen-induced RA rats.

In conclusion, our results demonstrated that magnolol mitigated the inflammatory response in TNF-α-stimulated human RA synoviocyte MH7A cell lines and CIA-induced RA rats, indicating that magnolol possibly serves as a potential treatment for RA. However, the current study has several limitations. First, the current study is This study represents an initial exploration of magnolol’s biological functions in osteoarthritis. However, several critical gaps remain before clinical translation can be considered [1]: comprehensive preclinical evaluation – including pharmacokinetics, safety profiling across major organ systems, and long-term toxicity assessment – is still required [2]; the precise molecular mechanism underlying magnolol’s anti-inflammatory effects in bone and joint tissues has yet to be elucidated. Further mechanistic and translational studies are therefore warranted to establish its therapeutic potential and inform future clinical development.
